# Accurate water quality assessment using IoNT-enabled deep learning frameworks

**DOI:** 10.1038/s41598-026-42563-3

**Published:** 2026-03-10

**Authors:** V. Rajakumareswaran, K. V. Uma, Sheshagiri Babu, N. Rajkumar

**Affiliations:** 1 Department of Computer Science and Design, Erode Sengunthar Engineering College, Thuduppathi, Tamil Nadu India; 2 Department of Information Technology, Thiagarajar College of Engineering, Madurai, Tamilnadu India; 3 Kakatiya Institute of Technology and Science, Warangal, Telangana India; 4 Vel Tech Rangarajan Dr.Sagunthala R&D Institute of Science and Technology, 600062 Avadi, Tamil Nadu India

**Keywords:** Internet of nano-things, WQ index, Nanosensors, WQ monitoring, Water management, Engineering, Materials science, Mathematics and computing, Nanoscience and technology

## Abstract

This work proposes a novel Internet of Nano-Things (IoNT)-driven real-time system architecture of water quality (WQ) observation and classification through a Convolutional Neural Network (CNN) framework, comprising WQI-CNN. The proposed system will be organized into four stages, namely, the data acquisition, coordination, data processing, and prediction and classification of the WQ Index (WQI). State-of-the-art nanosensors, such as Luminescent TOP, Surface Enhanced Raman Spectroscopy (SERS), and graphene-based sensors are used in the sensing phase to measure important WQ parameters. The data processing step uses Deep Generative Adversarial Networks (GANs) to fill in the gap between missing information and normalize data and improve the quality of predictions. WQI-CNN model incorporates these pre-processed inputs and uses CNN to create accurate WQI classification. The system was compared with the already existing systems such as the IoT-ML, WQI-ML, GTV-STP, which showed better performance in terms of computation time, RMSE, accuracy and MCC. The WQI-CNN model can be accurately used to determine the value of a real-time WQ monitor (98.91) which is essential in the management of the proactive water under the condition of the set of the safe drinking water standards.

## Introduction

Water is the main surface covering more than three quarters of the earth and all forms of life need it. Clean, edible, and abundant water is not available with its ubiquitous presence. Moreover, water is the usual carrier of numerous illnesses and thus real-time analysis of WQ is more than essential. Conventionally, assessment of WQ entails that samples taken in a large number of points are examined in laboratories. This is a manual procedure, which is not always efficient, expensive and time consuming. Smart monitoring systems are therefore being used more frequently to give real time information about WQ^1^.

Pakistan is also concerned with groundwater pollution^2^. Rapid modernisation, mining and agricultural practices have been very impactful to the groundwater reserves, especially in Punjab and Sindh. The use of these activities has contributed to contamination and low the value and volume of groundwater. The scenario has become aggravated in the last ten years because of increased human presence and ecological variability with the outcome of poor WQ and increased waterborne diseases^3^. WQ assessment is important to mitigate adverse impacts of human activities and environment transformations on water resources in the world^4^. The WQ indicators are chemical, physical, and biological parameters which are necessary in deciding the suitability of water to different uses. The WQI has been found to be useful in analyzing and determining WQ problems.

The WHO reports that drinking water that is not safe results in about 1.5 million deaths per year, the majority of them being infants and other young children^5^. Recent innovations in nanotechnology have created a motivation in creating nanosensors to monitor and treat pollution and as a way of offering creativity to the pollution issues. The integration of nanosensors into an IoNT between nanoscale devices and the IoT is one of the potential ways of how the IoT can be used in the future^5^. The nanosensor market is estimated to expand at 11.0 per cent CAGR between 2019 and 2026 projecting to reach USD 1.3 billion by 2026^6^.

The IoNT has lately demonstrated immense environmental monitoring capabilities whereby real-time data collection, transmission and processing can be achieved through the framework. IoNT systems by incorporating nanosensors with wireless communication networks provide the 24-hour monitoring of the environmental levels, including WQ indicators. The analysis of data collected by these nanosensors can be presented by the respective DL models, which will then forecast WQIs, which are required in evaluating the health of the water bodies.

This paper has presented a new-IoNT based model that incorporates nanosensors with a CNN model to forecast WQI, which has been named WQI- CNN model. The framework also utilizes the DeepGAN to manage missing data so that the CNN model can be trained utilizing full and reliable information. The WQI-CNN model overcomes the disadvantages of WQ prediction models, including those proposed by IoT-ML, WQI-ML, and GTV-STP, as it is more accurate, differs in fewer computation time and predictive potential. The given research brings some novelty to the process of WQ monitoring and prediction by incorporating the latest technologies, including IoNT and DL. The contributions of the work are:

- To improve the reliability of data inputs for the CNN model, the research incorporates a DeepGAN to impute missing or incomplete data, ensuring more accurate predictions.
- In order to ensure real-time decision-making in WQ management, the proposed model is intended to be used in integrating into IoNT systems, which will enable collecting data in real time and responding to WQ problems promptly.
- In a bid to enhance the performance of the model, feature correlation is done using the Spearman correlation that determines the most suitable WQ parameters so that the CNN can focus on the most significant features to give precise WQI forecasts.
- To obtain real time detection of problems, the solution proposal will be to integrate nanosensors into the IoNT, so that any abrupt alterations occurring in WQ can be identified and handled immediately to take fast actions regarding any possible occurrence of contamination.

The remaining portions of the work are arranged this way: The second part examines the relevant research and past research on the issue. The third part describes the materials and techniques, emphasising the IoNT-enabled structure for data gathering and the DL-enabled architecture for data analytics. This section includes discusses data preparation techniques and the DL architectures used. Section 4 evaluates the performance of the DL models in analysing and predicting WQ data. Lastly, Sect. 5 wraps up the work with a summary of the findings and a discussion of future directions.

The primary contribution of this study lies in the system-level integration of an IoNT-enabled sensing framework with a DeepGAN-based data imputation strategy and a CNN-based WQI classification model, rather than in proposing a novel standalone deep learning architecture. In contrast to other recent works which are based on the conceptual framework of ensemble machine learning models or the hybrid CNN-LSTM frameworks to predict the offline WQI, the current work highlights the single pipeline that underpins nanosensor-based data collection, missing-data distribution, correlation of features among features, and deep-learning-based classification.

The DeepGAN element is specifically used when dealing with incomplete and irregular sensor measurements, and this is a usual problem of the real-world IoNT implementations yet frequently ignored by other WQI forecasting studies. Besides, the proposed CNN model is tailored to effectively perform inter-parameter interaction of all water quality indicators with limited computational limitations hence applicable in near real-time analysis. This hybrid view separates the proposed framework with the existing WQI prediction frameworks that separate sensing, preprocessing, and learning as separate units.

### Related works

Ehteram et al.^7^ proposed a new hybrid model that is aimed at predicting the WQI with accurate precision. Their model is the CNN-CRNN-M5T, which is a combination of a CNN, a Clockwork Recurrent NN (CRNN), and an M5 Model Tree, to predict the WQ parameters. It is a model that can combine both space and time information effectively making WQ evaluations comprehensive. In comparison to other models, this model enhanced the Nash -Sutcliffe efficiency coefficient by 4–20% and 2.1–19%.

The research done by Latif et al.^8^ aimed to analyze the quality of drinking water in two Lahore metropolitan districts using physicochemical, WQI, and Specific Pollution Index (SPI) as measurement metrics.

The primary reasons for bad WQ were found as ageing water pipes, fast urbanisation, hazardous material seepage, incorrect waste management, and low groundwater levels in certain locations.

Sidek et al.^9^ suggested two ensemble and optimized ML models to predict the WQI of Johor River. Developing an ensemble ML model involved combining multiple individual ML models to enhance predictive performance. Rostam et al.^10^ provided evidence that choosing appropriate features and employing time series analysis with DL were significantly more effective in addressing the challenges connected with extremely irregular and changeable algal eco data. Predicting Harmful Algal Bloom (HAB) development was vital to water oversight, especially when constructing effective prediction models to reduce water contamination.

Hassan et al.^11^ evaluated the performance of ML methods, including RF, NN, MLR, SVM, and BTM, for predicting the WQ components of an Indian WQ dataset. A drawback of their work was the lack of DL approaches, which could have enhanced the efficacy of the selection process.

Wang et al.^12^ suggested a two-layer model stacking method for predicting seashore WQ. They integrated five commonly used methods into a single ML model. This stacked model was then fitted to 3 coastlines in eastern Lake Erie, New York, and compared to the separate base models. The model topping strategy surpassed all the basis algorithms, achieving consistently high accuracy with yearly averages of 78%, 81%, and 82.3% across the tested beaches.

Rahu et al.^13^ suggested an integrated framework that combined the IoT and ML for complete WQ investigation and forecast. However, the study’s drawback was its failure to incorporate DL techniques, which could have further enhanced the accurateness and trustworthiness of urban WQ investigation and forecast for cities.

Mathur et al.^14^ revised the several kinds of nanomaterial that had been used for eliminating poisonous metals from water. These nanomaterial possessed an extensive range of physical and chemical features, which made them a highly effective choice for waste treatment. Nishu and Kumar^15^ said that the development of innovative substances and methods allowed by nanotechnology has the potential to dramatically improve the efficacy and productivity of water filtration operations. Furthermore, improvements in nanotechnology-based detecting and tracking devices provided new options for live WQ identification and tracking. Continued study and advancement in nanotechnology for treating water was viewed as an interesting and promising way to tackling the worldwide problem of delivering pure and secure consuming water.

Senhaji et al.^16^ proposed a grouping of long-term and short-term memory IoT NN for real-time water observing. Their scheme was designed to screen WQ automatically, with updates made on website servers at a low cost and without requiring staff for service. Therefore, it was assumed that WQ analysis would be affordable and effective.

The available literature on the WQ monitoring and prediction has also noted the existing gaps to be addressed by the proposed model to a considerable degree. To begin with, integration of IoT-ML models has been a problem in terms of real-time data integration and this forms a constraint to the timely responses and insights to WQ change^13^. The consequence of this problem is the inactive water management practices. The suggested IoNT-based framework is able to surmount this constraint by investigating the integration of a network comprising linked nanosensors enabling real-time and continuous monitoring and instantaneous information transfer. Secondly, the WQI-ML model has been criticized to be deficient in several parameters of WQ like BOD and absence of the correlation analysis among various parameters^17^. This exclusion is a weakness in the model in offering a holistic evaluation of WQ. The proposed model will fill this gap by combining these important parameters and conducting an extensive correlation analysis.

As well, GTV-STP models have been found not to be effective in understanding the true distribution of data because significant information is eliminated^18^. This weakness has impacts on the generalisation capacity of the model as well as accuracy of the prediction. The proposed model helps in addressing this problem as advanced nanosensors are being used to record detailed and high-resolution data, which is processed by a heavy CNN architecture.

## Methodology

In the proposed IoNT-based framework, four phases, which include the sensing phase, coordinator phase, data processing phase and the WQI prediction and classification phase are designed. Each of the phases can be described by the following functions.

The data sample in this work entails multivariate measurement of water quality that has been obtained via an IoNT-based sensing system that was implemented on several freshwater monitoring stations. The obtained data are real sensor values of nanosensor-enables nodes, which sense physicochemical and biological parameters, such as temperature, pH, dissolved oxygen, (DO), electrical conductivity, biological oxygen demand (BOD), nitrate concentration, fecal coliform, and total coliform.

They sampled on a regular time schedule and recorded data in a tabular form with each row of the table fielding one sampling moment and each column fielding a certain water quality parameter. According to the data, there was a total number of N samples that came together at K locations of monitoring at a period of the T months.

The validation of raw sensor data was performed to eliminate physically invalid values to guarantee the reproducibility of results and then normalized with normalization that was accomplished by operating the DeepGAN-based imputation strategy. The overall dataset was the one which has been randomly split into 80% training data, and 20% testing data, such that all monitoring locations have been covered in both sets.

The processed data is accessible to the respective author on reasonable request, which makes it possible to verify the experiment results independently and reproduce them.

### IoNT enabled data acquisition: sensing phase

The surveillance process involves the establishment of many physical, chemical and biological factors. The information is collected periodically at different locations. The number of monitoring the water supply is based on the factors of its importance, legal requirements, and the available resources. Besides the human demanding process of collecting water samples, this research places a proposal of an IoNT solution to monitoring the WQ. The proposed solution would involve automated data precising through networked gadgets and sensors. The little sensors would constantly monitor the attributes of temperature, dissolved oxygen (DO), pH, conductivity, biological oxygen demand (BOD), nitrate, and fecal coliform and total coliform. This obtained information would be wirelessly relayed to a central unit where it would be processed and stored in real-time. The proposed IoNT-based system would provide prompt and quality WQ data, and quick response and successful monitoring of water conditions.

- **Luminescent TOP Nanosensor**: An advanced nanosensor called Luminescent TOP^19^ nanosensor has been observed to measure temperature (“T”), oxygen (“O”), and pH (“P”) simultaneously using optical tools.
- **SERS**: These nanosensors^19^ are very sensitive devices utilized in detection of the existence of fecal coliform bacteria in water. The nanosensors use metallic nanoparticles (usually silver or gold) that increase the Raman scattering signal of target molecules when these are present in fecal coliform bacteria it is possible to detect even the bacteria that have very low concentration.
- **Ion-Selective Nanosensors and Graphene-Based Nanosensors**: Ion-Selective Nanosensors will have nitrate-specific membranes to identify nitrates by utilizing a unique response to changes in the electrical characteristics of the sensor, and these will give the real nitrate concentrations. Graphene-Based Nanosensors^19^ on the other hand uses graphene with its outstanding electrical conductivity to detect fluctuations in electrical resistance that are linked to ionic level of concentration of water effectively determine conductivity.

It is to be noted that nanosensors utilized in this structure are founded on either well-known sensing conceptions that have been documented in the latest literature as opposed to custom-made hardware prototype models that have been created in this research. The Luminescent TOP nanosensor, SERS-based biosensors, ion-selective nanosensors, and graphene-based conductivity sensors have been previously validated for environmental monitoring applications, as reported in^6,19^, and related studies.

In the present work, these nanosensors are considered as representative sensing modalities within an IoNT architecture to demonstrate the feasibility of real-time, high-resolution water quality monitoring. The focus of this study is on system-level integration and data analytics rather than on nanosensor fabrication or calibration, which remain outside the scope of this manuscript.

### Coordinator phase

The coordinator phase manages and orchestrates the various tasks and components within the system, effectively linking the sensing phase to the data processing module. This phase is outfitted with an Arduino control and LoRa connectivity^1–5,20,21^. The Arduino control system is the core device that collects data from the sensors during the detection phase, while the LoRa protocol enables distant wireless connectivity, allowing the Arduino to communicate the acquired data to the next step.

### Data processing phase

Deep GAN is employed to fine-tune input data to ensure a high level of prediction and prevent a loss by preprocessing this data using a set of missing value imputers, and scaling the features to normality. The data is then preprocessed and divided into two parts; 80% training and 20% testing. Nanosensors that record WQ parameters have missing value parameters that are handled through the use of a GAN model that imputes the data by employing spatial and temporal relationships. The GAN system comprises of a generator and a discriminator, which learns to adapt imputed values such that their results look like real values.In the suggested model for WQI prediction, consider a collected dataset represented as$DS={\left\{{w}_{1},{w}_{2},\dots{w}_{n}\right\}}^{k}$ where K represents the total number of sampling points. Suppose the masking vector ${m}_{v}$ is used to represent the presence (1) or absence (0) of data, indicating missing values. The incomplete dataset is then defined as$\stackrel{\sim}{DS}={\left\{{\stackrel{\sim}{w}}_{1},{\stackrel{\sim}{w}}_{2},\dots{\stackrel{\sim}{w}}_{n}\right\}}^{k}$, where each $\stackrel{\sim}{w}$ is a random variable masked by ${m}_{v}$, with missing data denoted as:

1 $${\stackrel{\sim}{w}}_{x}=\{{w}_{x},if{m}_{vx}=1nan,if{m}_{vx}=0$$

In this model, the generator uses the samples${\stackrel{\sim}{w}}_{x}$, noise $N$, and masking vector ${m}_{v}$ to generate imputed data. The imputed value vector $\underset{\_}{w}$ is computed using the generative network, denoted as:

2 $$\underset{\_}{w}=g(\underset{\_}{w},{m}_{v},(1-{m}_{v})\otimes N)$$

Where $g$ represents the operation in the network. The final imputed dataset,$\widehat{w}$ is obtained by combining the observed data and the imputed values, such that:

3 $$0000\widehat{w}={m}_{v}\otimes \underset{\_}{w}+,(1-{m}_{v})\otimes\widehat{w}$$

 

The element-wise multiplication of matrices is performed using the operator$\otimes$. The architecture of the GAN in this model includes two fully connected layers. The activating units utilised in the hidden and final layers are ReLU and sigmoid, correspondingly. The mathematical actions of these activation functions are defined as follows:

4 $$Relu:f\left(y\right)=\left(0,y\right)=\{{w}_{x},if{w}_{x}\ge10,if{w}_{x}<0$$

5 $$Sigmoid:f\left(y\right)=\frac{1}{1+{exp}^{-x}}$$

The quality of imputation is ensured using a hint method, which provides partial information about missing data to the discriminator. This method involves using a hint matrix that combines imputed data with observed data, where the hint matrix marks missing data as 0 and observed data as 1. The discriminator attempts to predict this mask matrix, distinguishing between missing and real data. This is an adversarial training method which guarantees good imputation. To assess the variances of the data between the actual and the forecast value, the binarial cross-entropy value is adopted that improves the accuracy of the imputed data. The hint matrix directs the discriminator in predicting the mask matrix and the result is successful imputation even in case of substantial quantities of missing data. The objective function is defined as:

6 $$minGenmaxDis{E}_{\widehat{w},{m}_{v},H}[{m}_{v}^{T}log\widehat{{m}_{v}}+{\left(1+{m}_{v}\right)}^{T}loglog\left(1-log\widehat{{m}_{v}}\right)]$$

The term “log” refers to the element-wise function, and ${m}_{v}$ represents the projected mask matrix, denoted as${m}_{v}=DS(\widehat{w},H)$. The binary cross-entropy loss operation is described as the negative log of the correct forecasted likelihood.

7 $$L\left(a,b\right)={\sum}_{i=1}^{n}[{a}_{i}loglog\left({b}_{i}\right)+(1-{a}_{i})log(1-{b}_{i}\left)\right]$$

8 $$minGenmaxDisE\left[L\left({m}_{v},\widehat{{m}_{v}}\right)\right]$$

**Fig. 1:**
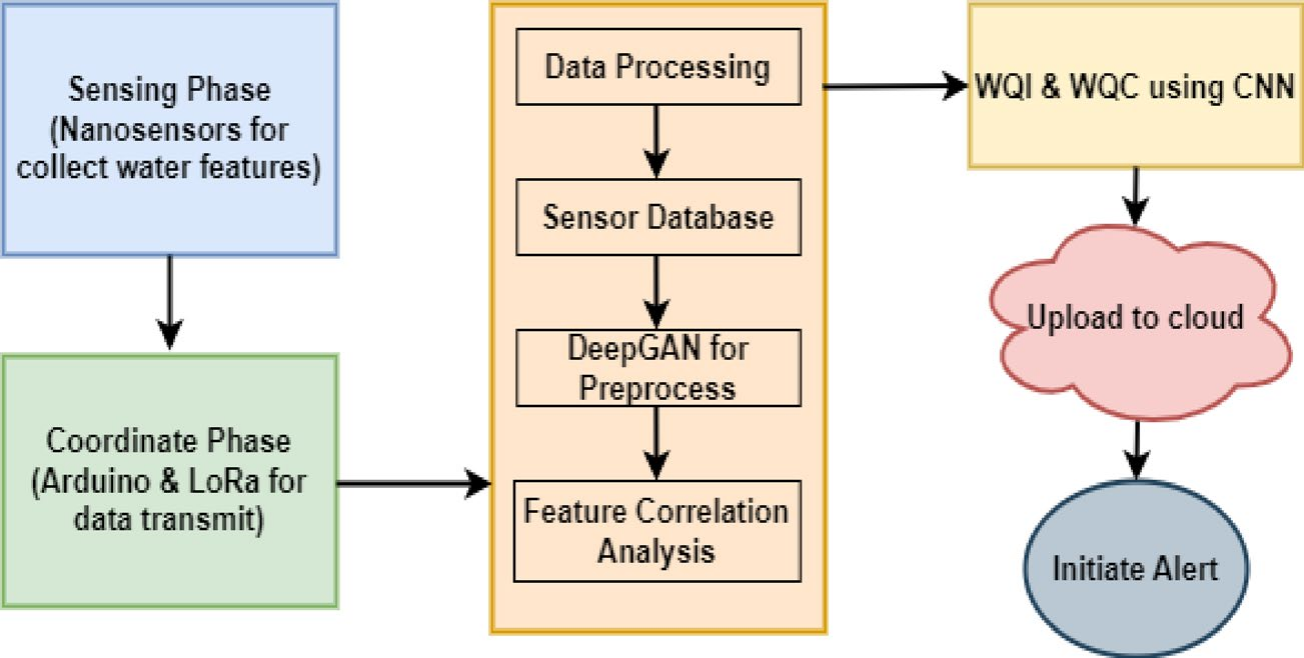
Suggested work for WQI prediction and classification.

This operation is made easier and applied to enhance the imputation of missing data using GAN, which guarantees that there is proper data pre-processing prior to the implementation of regression to make predictions. As shown in Fig. 1, the architecture of proposed work is in the follow up.

### Finding feature correlation

The correlation factor is a statistic used to estimate the significance of the relationship between two factors and it is also used to indicate how much the change in one variable is associated to the change in another. As shown in Table 1 below. In the case of WQ analysis, the Spearman correlation coefficient especially comes in handy because it is used to quantify the strength and direction of the relationship between two ranked variables.

Its calculation formula is as follows:

9 $$corr=\frac{{\sum}_{i}^{}({a}_{i}-\underset{\_}{a})({b}_{i}-\underset{\_}{b})}{\sqrt{{\sum}_{i}^{}{({a}_{i}-\underset{\_}{a})}^{2}{{\sum}_{i}^{}({b}_{i}-\underset{\_}{b})}^{2}}}$$

**Table 1:** Division of correlation.

Values	Correlation
0.8–1.0.8.0	Very strong
0.6–0.8	Strong
0.4–0.6	Moderate
0.2–0.4	Weak
0.0–0.2.0.2	Very weak or not correlated

As an illustration of this, when evaluating the relationship between DO and BOD, a strong negative correlation would be found meaning that, the higher the BOD, the lower the DO would be likely to be. Likewise, the pH parameters possibly convey a linear relationship with the conductivity with variations in either of them being used to forecast another.

Before proceeding the imputation or normalization steps, the dataset was first broken into training (80 per cent) and testing (20 per cent) chunks to avoid information leakage. DeepGAN-based missing data imputation model was also only trained on the training subset and after that the trained generator was used to impute missing values in test data. This makes sure that there was no information regarding the test set that was used to influence the model training or preprocessing that would affect the validity of evaluation protocol.

### WQI prediction and classification phase

The WQI is an important indicator that is employed to assess WQ^22^. It is calculated by adding a number of parameters that depict different attributes of WQ. The WQI is determined by the following formula:

10 $$WQI={\sum}_{i=1}^{n}\frac{{q}_{i}\times{w}_{i}}{{w}_{i}}$$

Here, $n$ denotes the quantity of variables, ${q}_{i}$ represents the effectiveness rating of variable$i$, and ${w}_{i}$ is the unit weight assigned to parameter. The ${q}_{i}$ determined using:

11 $${q}_{i}=\left(\frac{{e}_{i}-{v}_{i}}{{s}_{i}-{v}_{i}}\right)\times100$$

Where ${e}_{i}$is the witnessed value of variable, ${v}_{i}$ is the ideal value when the water is pure, and ${s}_{i}$ is the standard value for variable. The ${w}_{i}$ calculated as:

12 $${w}_{i}=\frac{c}{{s}_{i}}$$

Where $c$ is a proportionality constant and is computed as:

13 $$c=\frac{1}{{\sum}_{i=1}^{n}{s}_{i}}$$

The proposed model with CNN architecture is effective in learning complicated patterns and spatial relationships of WQ data and this is important to properly classify WQ scenarios. According to this model, the features that have been taken by nanosensors are initially preprocessed to address the absence of data and normalization of values. These preprocessed features are then used to train the classification model. The input layer of the CNN processes the data, which is then passed to the hidden layers^23^. Every hidden node in these layers applies weights to the input values, contributing to the network’s learning. The final output layer uses the softmax function to produce probabilities for different WQ classes based on the WQI values. The features and their weights are expressed ${I}_{j}={I}_{\mathrm{1,2},3.....n}$and${W}_{j}={W}_{\mathrm{1,2},3.....n}$. Compute the inputs that are selected, by the weight vectors, and then add them together:

14 $$S={\sum}_{j-1}^{n}{I}_{j}{W}_{j}$$

Where the cumulative value is denoted by$S$. Define the activation function.

15 $${AF}_{j}={R}_{j}\left({\sum}_{j-1}^{n}{I}_{j}{W}_{j}\right)$$

16 $${R}_{j}=exp(-{I}_{j}^{2})$$

Here, ${AF}_{j}$implies the activation function, whereas ${R}_{j}$indicates the exponential of${I}_{j}$. Compute the outcome of the next hidden layer using,

17 $${H}_{j}={b}_{j}+{\sum}_{}^{}{R}_{j}{W}_{j}$$

Where ${b}_{j}$ indicates the value of bias and ${W}_{j}$defines the relationship among the input layer and hidden layers in terms of weight. The three processes above are repeated for all layers of CNN. In the end, the output value will be assessed by combining the weights of all inputs to get the values of the neurons of the output layer:

18 $${G}_{j}={b}_{j}+{\sum}_{}^{}{M}_{j}{W}_{g}$$

Where ${M}_{j}$ represents the layer value before the output layer, ${W}_{g}$defines the hidden layer’s weights, and ${G}_{j}$denotes the final unit. The output with the target outcome is compared in this phase. The error signal is the difference between these two numbers. This value is represented numerically as

19 $${E}_{s}={T}_{j}-{G}_{j}$$

Where ${E}_{s}$indicates the error signal and ${T}_{j}$denotes the target outcome. The output rate is compared to the desired value. The error associated with it is identified. A value ${Z}_{j}$is generated based on this error, and then used to send the error to the output units in the network.

20 $${Z}_{j}={E}_{s}\left[f{(G}_{j}\right)]$$

The back-propagation technique is used to perform the weight adjustment. This relationship is as follows:

21 $${W}_{cj}={PZ}_{j}{(I}_{j})$$

In the above equation, ${W}_{cj}$denotes the weight alteration, $P$ specifies the momentum, and ${Z}_{j}$ is the error that is transmitted throughout the network.

**Table 2:** WQ categories.

WQI	Quality
0–25	Excellent
26–50	Good
51–75	Poor
76–100	Very poor
> 100	Unfit for drink

The categorization is useful in indicating the general condition of WQ according to the projected WQI. By examining the uploaded data and forecasting the WQI values into cloud, should the response indicators indicate that the WQ occupies either the poor or very poor or unfit to consume category (as illustrated in Table 2), the system will be capable of provoking necessary actions. This can involve sending alerts to concerned parties so that they can take the required remediation measures.

The WQI-CNN model has been suggested to be executed based on tabular time-indexed water quality data that are re-structured into a two-dimensional form (tensor) to facilitate convolutional feature extraction. The inputs are expressed as a fixed-length feature vector intending every sample of the input, which is the value of the normalized water quality parameters at a specific time point.

The CNN is an architecture which has an input layer and three convolutional layers containing 3 × 1 kernel size and used ReLU to activate functions. To maintain the dimensionality of features, zero-padding is employed and after every block of convolution max-pooling layers are employed to reduce computation, as well as, help the model to avoid overfitting. A dropout rate of 0.3 is used to increase the performance of generalization.

The resulting extracted feature maps are flattened and sent through two fully connected layers, and finally a softmax output layer that makes a predetermined classification of the forecasted WQI into predefined quality classes. The indicated model is trained by means of the Adam optimizer coupled with the categorical cross-entropy as a loss measure and a learning rate of 0.001.

The use of CNNs as compared to recurrent architectures like LSTM or Transformer models is because they are better at capturing local feature interaction between correlated water quality parameters with lower computational cost, which is more applied to real-time monitoring with the ability to be run on an IoNT network.

## Performance evaluation

**Fig. 2:**
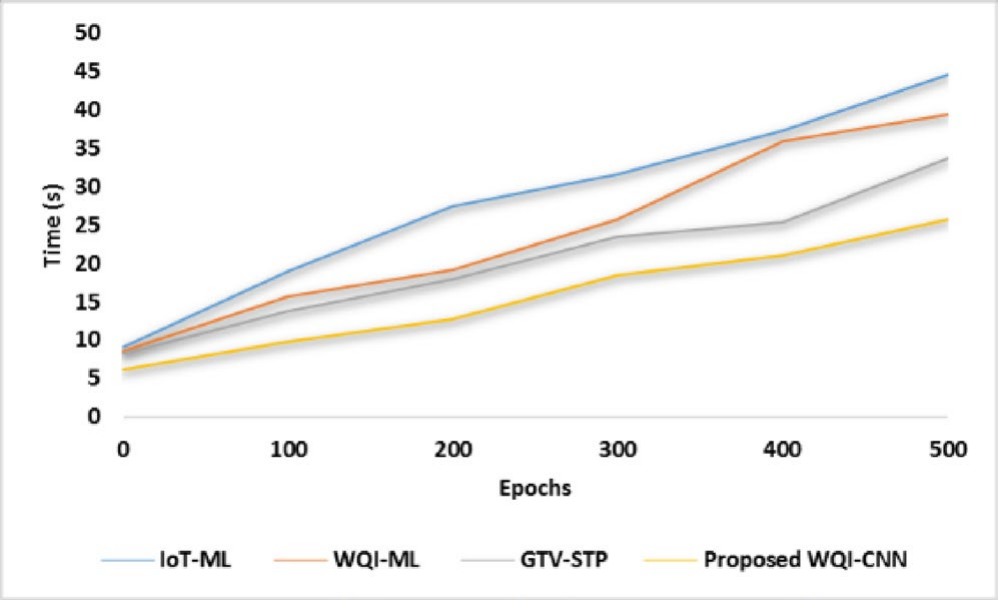
Computation time.

Each of the baseline models (ioT-ML, WQI-ML and GTV-STP) was re-implemented and tested with the same dataset, preprocessing pipeline, feature set, and traintest split used with the proposed WQI-CNN model to keep the comparison fair. The hyperparameters of each of the baseline models were chosen variously in accordance with the suggestions of the original literature and were also developed according to the current dataset. The requirements of the experimental setup are as follows: the Intel(R) Xeon(R) 8272CL CPU at 2.60 GHz and 32 GB of RAM and the windows 10 64-bit. The NN system was developed based on Python 3.9.6, Keras and TensorFlow 2.6.0 with Visual Studio Code IDE. Figure 2 below compares the computing time of four other models, which are IoT-ML^13^, WQI-ML^17^, GTV-STP^18^, and the proposed WQI-CNN as different epochs. The number of epochs to calculate the computations varies with the model, but its results vary significantly between the models. The IoT-ML model has the longest to compute time of all epochs as the minimum computation time is 9.1s at 0 epochs and the maximum is 44.6s at epoch 500. This means that it is the most inefficient in computations as compared to other models that have been compared. The failure to combine real-time data is also the main limitation of the IoT-ML model that prevents the development of intellectual decision support schemes. Such a limitation may result in less active and efficient practices of water organization, which can enhance its increased computation times.

The suggested WQI-CNN architecture keeps showing the minimal time of computation in every epoch. It begins at only 6.2s at 0 epochs and only Hits 25.7s at 500 epochs only the most efficient model with regard to the computation time. WQI-CNN model is created to effectively handle and analyse real time data available through nanosensors hence important to the correct and prompt WQ prediction.

**Fig. 3:**
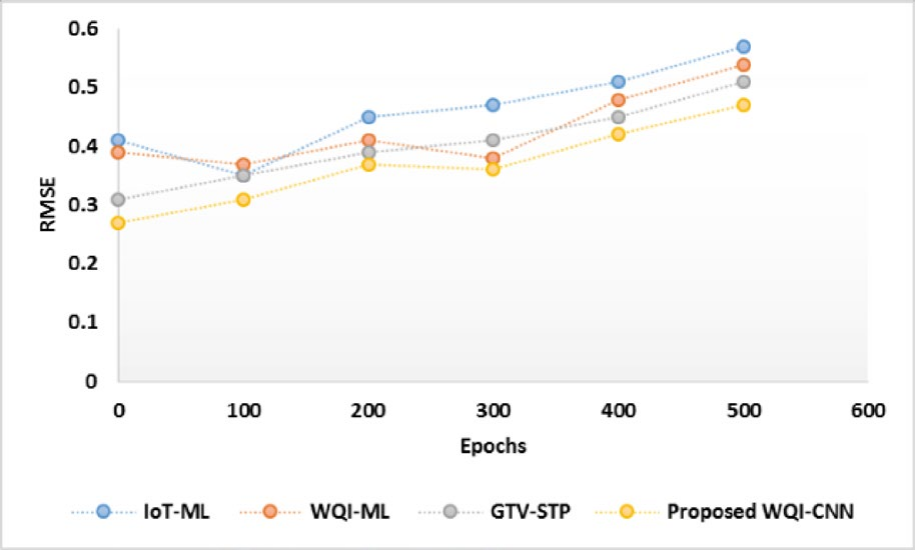
RMSE comparison.

Figure 3 presents the RMSE values of the various models after a number of epochs and this clearly demonstrates the level of accuracy and stability of each model in terms of its predictions. WQI-ML model has a lower starting RMSE of 0.39 at epoch 0, indicating high initial accuracy, than the IoT-ML. The fluctuation rate of the RMSE is small but generally increases at the end of the epochs reaching 0.54 at 500 epochs. This aspect implies that the WQI-ML model is initial more stable, but its overfitting behavior occurs over time during training. The initial accuracy is better with the proposed WQI-CNN model, the RMSE is 0.27 at epoch 0, which is lower than the initial value in the newly proposed model. General error reduction of about 17.54% over IoT-ML, 12.96% over WQI-ML and 7.84% over GTV-STP.

Proposed WQI-CNN model successfully addresses the negativities of the WQI-ML model, which include the omission of key WQ variables, such as BOD, as well as, failure to provide correlation analysis of various WQ variables. The WQI-CNN model is more comprehensive and accurate in the evaluation of WQ by including all the vital parameters.

The use of BOD is especially important since it is one of the indicators of organic pollution and WQ. Besides, the proposed model includes an elaborate correlation analysis that enables it to facilitate the associations among the various water parameters. It is also able to not only increase the predictive accuracy of the model, but also to simplify to unseen data, which means that the WQI-ML model has drawbacks in offering a complete and dependable WQ index.

**Fig. 4:**
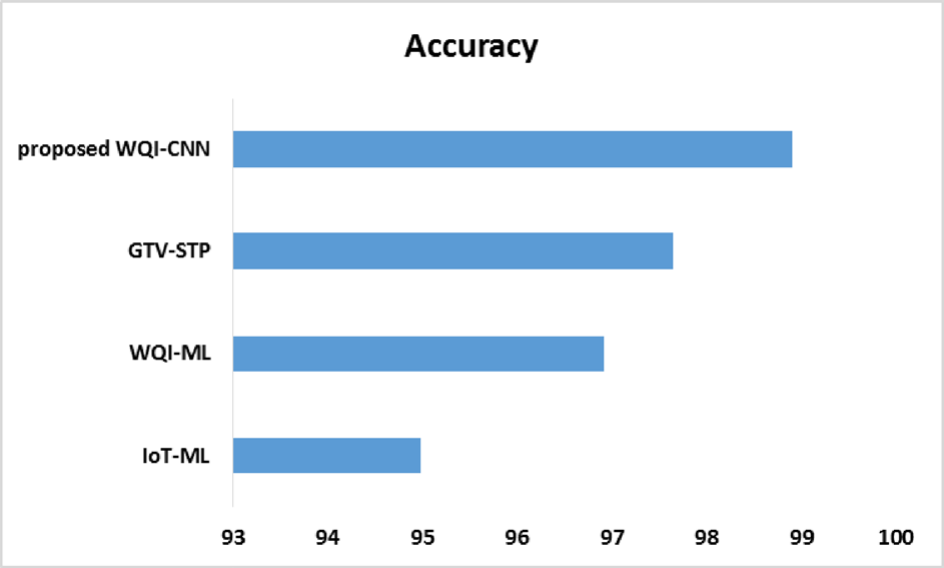
Accuracy comparison.

The comparison presented in Fig. 4 of the evaluation of accuracy during the models indicates the better performance of the proposed WQI-CNN model in predicting WQ. The WQI-CNN model is the most accurate with an accuracy of 98.91 than the other models, that is, in IoT-ML, WQI-ML, and GTV-STP the percentages are 94.98, 96.92 and 97.65, respectively. This is of significant interest, and the WQI-CNN model has experienced an increase in accuracy by 3.93% compared to IoT-ML, 1.99% compared to WQI-ML and 1.26% compared to GTV-STP.

GTV-STP model has a great weakness of omitting certain important portions of the information that inhibits its capacity to depict the true spread of the information. Such an omission has a direct impact on the generalization ability and prediction power of the model, such that it is less effective in varying situations. The suggested WQI-CNN model makes amends this disadvantage by implementing a full range of features and advanced neural network topology, which are strong at learning intricate forms and association on the data. Through the use of CNNs, the recommended model is able to effectively analyze spatial and time-related dependencies that are vital in the effective prediction of WQ.

**Fig. 5:**
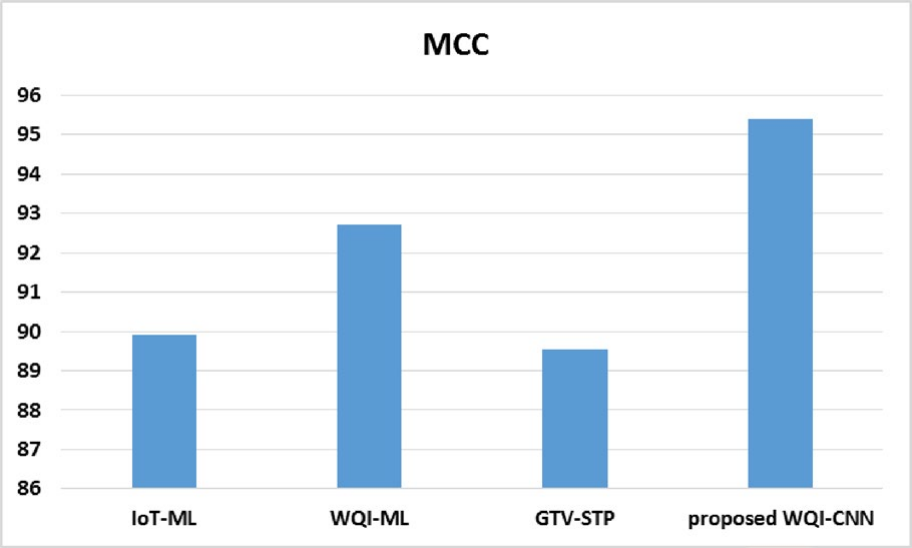
MCC analysis.

The MCC (Fig. 5) is a quality measure of the quality of classifications and this reflects the balance of various types of errors of classifications. The proposed model WQI-CNN has the greatest MCC value of 95.4% which means that the model has a better performance in terms of the performance of the classification as well as the reliability with the existing frameworks. The MCC of WQI-ML model is 92.71 which is heavy, nevertheless, less than the performance of the recommended model. The predictive power and balance in GTV-STP and IoT-ML is even less with the classifications yielding MCC values of 89.54% and 89.9% respectively.

The better value of MCC in the proposed WQI-CNN positively indicates that it is more effective to forecast the WQI within minimizing the false positives and false negatives rates. This enhancement is especially important in the sense that increasing MCC value is generally associated with more accurate forecasts which is where it is important that equal accuracy can be accomplished which applies to be very essential when making dealings as well as WQ management.

**Fig. 6:**
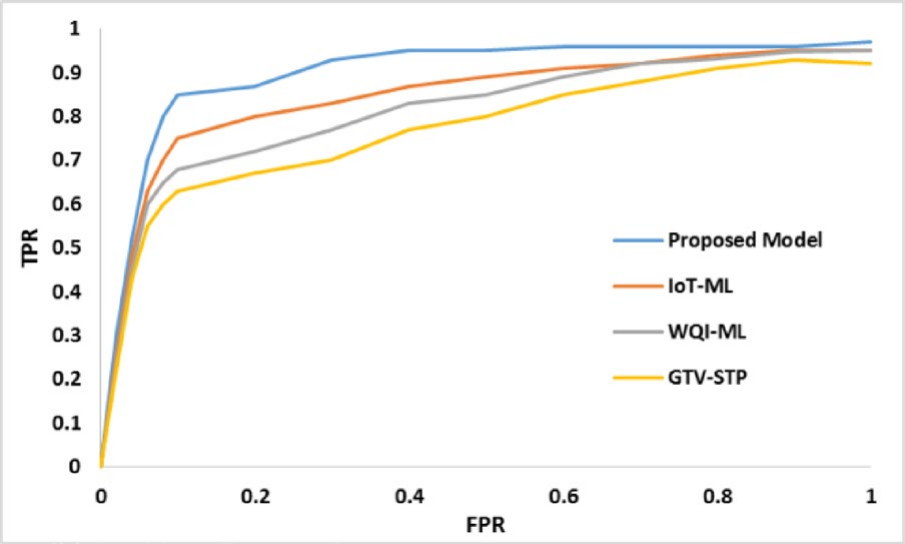
ROC analysis.

The comparison of The ROC (Fig. 6) shows that the proposed WQI-CNN model is better in testing using the True Positive Rate (TPR) at different stages of the False Positive Rate (FPR) compared to the other existing models (IoT-ML, WQI-ML, and GTV-STP). As an example, the TPR obtained with the proposed model (0.3) is a little bit better than IoT-ML (0.22), WQI-ML (0.26), and GTV-STP (0.24). When the FPR is increased to 0.5, the proposed model is the one that has the TPR of 0.95, followed by that of IoT-ML, WQI-ML, and GTV-STP at 0.89, 0.85, and 0.8, respectively. This remains the case and the proposed model still has a constant TPR of 0.97 even at increased FPRs, which is greater than the other methods.

The increased performance of the proposed WQI-CNN model could be explained by the following important aspects, such as utilizing DeepGAN to preprocess the data and combining nanosensors to capture real-time data. DeepGAN is used to fill in missing values in the dataset based on spatial and time relationships and this has helped a lot to improve the quality of input data. DeepGAN also solves the issue of information gaps and discrepancies exceeding the boundary of the deep learning network, making training the model on incomplete and unreliable data unattainable and thus inaccurate prediction and categorization.

Furthermore, nanosensor application is also significant in the process of recording high resolution data on different WQ parameters in real-time. Such a real time data collection will enable the model to analyse and predict WQI with every bit more accuracy and in real time. High-quality input data and real-time data processing allow the suggested WQI-CNN model to surpass the traditional models as the analysis of the ROC curve demonstrates.

Even though the proposed WQI- CNN model has shown superiority over the three models of IoT- ML, WQI-ML and GTV- STP, some limitations are purported to exist. The existing experimental testing is based on the information gathered at a few monitoring sites and there was no explicit cross-location generalization evaluation. Prediction accuracy could also be affected by season, sensor drift over the long term and extreme environmental events.

Further research will be conducted to evaluate the benchmark model by including other models (Random Forest, XGBoost, Multilayer Perceptron, and Long Short-Term memory); each framework will be tested with the other because the evaluation shall be on different geographical areas and seasons to further compare and test the resilience and the generalization capacity.

The documented performance measurements are the case of deterministic experimental tests applying constant random seeds. Although the results obtained have shown performance improvements relative to the baseline models across the study, the current study did not perform variance estimation as well as formal testing of statistical significance.

The further refinements of the coming needs to carry out repetitive experiments including numerous random initializations and reporting the mean and standard deviation values of each of the performance measurements and performing the necessary statistical tests to see whether the performance improvements are even more confirmed.

## Conclusion and future direction

The suggested WQI-CNN framework, which is based on IoNT, is an important development in the sphere of WQ monitoring and prediction. This system gives a real-time and effective method of measuring WQ through the incorporation of nanosensors, DL, and real-time data processing. Application of the sophisticated nanosensors is capable of processing the continuous monitoring of different variables in WQ, whereas DeepGAN model manages missing data well, such that the predictive model is trained only on a full and trustworthy dataset. The higher performance of the WQI-CNN model in several indicators, such as computation time, RMSE, accuracy (98.91%), MCC, and ROC, proves their effectiveness in predictive and classification of WQ indices, being superior to the other current models of the same type, including IoT-ML, WQI-ML, and GTV-STP. The suggested framework provides a better precision besides strengthening the decision making process in WQ management through timely alerts related to poor water conditions. More sophisticated sensors that could then be integrated into the WQ in the future will offer an even better evaluation of the WQ, i.e., be able to detect even the emergent contaminants e.g., microplastics or pharmaceuticals.

While the proposed framework is designed with real-time monitoring objectives in mind, the current evaluation is limited to simulation-based analysis of sensor data and algorithmic performance. System-level factors such as communication latency, sensor sampling rates, energy consumption, and deployment-specific constraints were not explicitly evaluated. Therefore, the presented results should be interpreted as demonstrating analytical feasibility rather than fully deployable system performance.

## Data Availability

The datasets used and/or analysed during the current study available from the corresponding author on reasonable request.
